# Prevalence and Clinical Types of Tremor in Multiple Sclerosis and its Associated Disability: A Systematic Review

**DOI:** 10.5334/tohm.776

**Published:** 2023-09-13

**Authors:** Prajjwol Luitel, Nischal Neupane, Sujan Paudel, Niranjan Adhikari, Binita Timilsina, Anil Suryabanshi, Prakash Gyawali, Rajeev Ojha

**Affiliations:** 1 Maharajgunj Medical Campus, Tribhuvan University Institute of Medicine, Maharajgunj 44600, Kathmandu, Nepal; 2 Emergency Department, Sukraraj Tropical and Infectious Disease Hospital, Teku 44600, Kathmandu, Nepal; 3 Department of Neurology, Tribhuvan University Institute of Medicine, Maharajgunj 44600, Kathmandu, Nepal

**Keywords:** tremor, multiple sclerosis, systematic review

## Abstract

**Objective:**

To describe the state of literature regarding prevalence, clinical types of tremor in Multiple Sclerosis and associated disability.

**Background:**

Tremor has long been recognized as an important symptom of multiple sclerosis. This can be intention and postural tremor that affects the upper limbs. Patients with multiple sclerosis who experience tremor of any severity typically retire early or lose their jobs due to disability.

**Methods:**

This systematic review was performed up to September 9, 2022. Article selection was performed by searching the MEDLINE (PubMed) and EMBASE electronic bibliographic databases. The search strategy was not limited by study design but only for articles in the English language.

**Results:**

A total of nine full-text articles were included in the analysis. Six studies were cross-sectional studies; one each was a prospective observational study, a case-control study, a community-based cohort. The prevalence of tremor in the multiple sclerosis (MS) population among studies ranged widely, between 12.5% and 68.9%. The presence of severe tremor ranged from 3% to 33%. Younger age was a significant predictor of tremor in two studies. The most common tremor subtype was action tremor. Upper extremities were the most common site involved in the majority of our studies, followed by head and neck.

**Conclusions:**

Prevalence of tremor ranged from 12.5% to 68.9% in the MS population with severe tremor being an infrequent complication. Severity of tremor correlated with increasing disability. Upper limb action tremor was the most common with rare occurrences of resting and rubral tremor.

## Introduction

The prevalence of movement disorders in multiple sclerosis (MS) is traditionally assumed to be low [1]. However recent studies have shown that movement disorders are not uncommon in MS, even early in the disease course. Approximately one third to half of individuals with MS may experience the development of tremor at some point during the progression of their disease [2]. Tremor in MS is typically bilateral, affects the upper limbs more than the lower limbs, but can also impact the head, neck, and even the vocal cords [2,3,4]. Patients with MS commonly experience intention and postural tremor [2]. MS patients with tremor of any severity modify their daily activity, retire early or become unemployed because of disability. Failure to identify MS as a potential cause of new-onset tremor can lead to delays in diagnosis and the beginning of disease-modifying therapy [2].

The exact prevalence and incidence of tremor in MS is challenging to determine because of variability in tremor presentation in terms of types, severity, frequency, evolution, progression; overlap with other symptoms like spasticity, ataxia, weakness; variability in reporting of symptoms depending on activity level of the patient. Current data are based on retrospective or small case series or few prospective articles which differ in clinical characteristics [5].

The aim of this article is to systematically review all the existing studies to comment upon prevalence of tremor, clinical types in multiple sclerosis and associated disability.

## Materials and Methods

**Figure 1:**
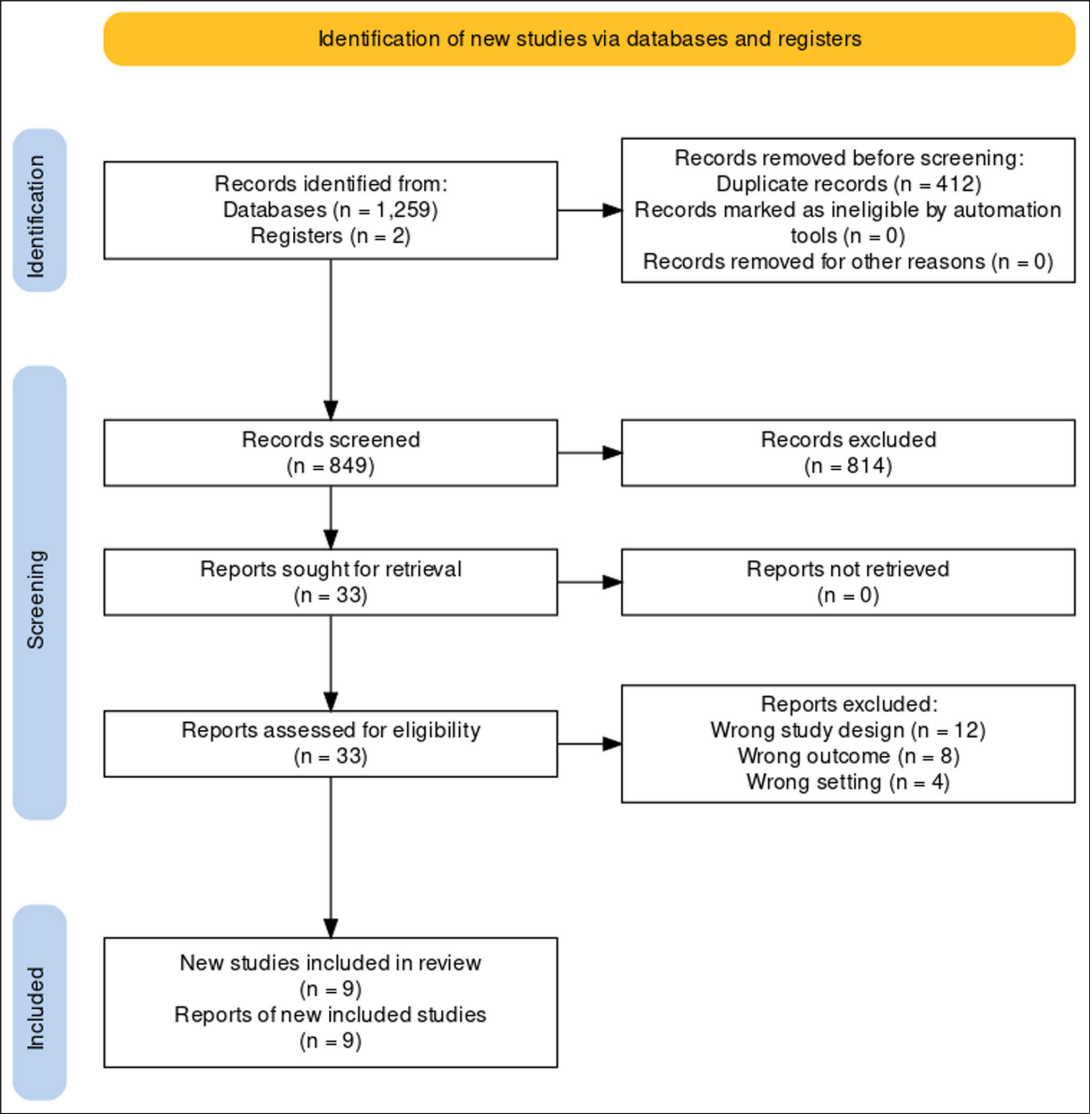
PRISMA diagram illustrating the study retrieval process.

The present systematic review was conducted in adherence to the Preferred Reporting Items for Systematic Review and Meta-Analysis (PRISMA) guidelines [6] (Figure 1). Screening was performed for all articles published up to September 9, 2022.

### Information source and search strategy

References were identified from searches of PubMed using a search strategy (found in supplementary file section) by two authors (PL, NN) independently. For each study shortlisted via this process, the reference section of the paper was checked to identify further studies not found in previous database searches. Furthermore, unpublished studies were searched in gray literature.

### Inclusion criteria

Cross-sectional, case control, and prospective studies reporting cases, prevalence, or incidence of tremor in MS with n > 2 patients published in the English language as full texts or abstracts were considered eligible to be included in this review. If two or more studies, included the same set of patients we included the study with the greater sample size.

### Exclusion criteria

Case reports, systematic review/meta-analyses, editorials, viewpoints, commentaries; articles with participants other than MS patients; missing/insufficient data; irretrievable (articles in other languages and non-accessible) studies; and MS studies reporting movement disorder except for tremor were excluded.

### Study selection and data extraction

1261 studies were retrieved for screening from the search databases, and duplicate records within bibliographic databases were removed. The remaining studies were screened by two authors (PL, NN) for title and abstract. The two screening authors individually performed full-text reviews of selected studies based on inclusion and exclusion criteria using COVIDENCE. Nine studies were finalized for review. Any discrepancies were resolved by consultation and discussion. Finally, relevant data from the selected studies were extracted into an Excel spreadsheet under specific headings (ID, Title of Study, country, study design, population characteristics, duration of MS diagnosis, MS pattern, median Expanded Disability Status Scale [EDSS], total number of patients with tremor, type of tremor, site of tremor, severity of tremor).

### Risk of bias assessment

The quality of the studies was assessed individually by 2 authors (PL, NN) using the “Joanna Briggs Institute (JBI) Critical Appraisal Checklist for cohort studies” for observational studies, the “JBI Critical Appraisal Checklist for case-control studies” for case-control study and the “JBI Critical Appraisal Checklist for cross-sectional studies” for cross-sectional studies. 11 questions were used to assess the quality of cohort studies, 10 questions for case-control study, 8 questions for cross-sectional studies. The following questions were dealt with: the selection of groups the assignment of exposures to groups, the measurement of exposures, the identification and management of confounding factors, the presence of outcomes at the beginning, the measurement of outcomes, the reporting of follow-up time, the sufficiency of follow-up, the strategy to deal with incomplete follow-up, and the statistical methods employed. The consensus was made between the authors to label the risk of bias according to the score assigned by the JBI scale.

### Summary Measures

Prevalence or cases of tremor in MS were used as the primary effect measures. Site, type, severity, tremor-related disability, predictors of tremor were secondary effect measures. For the studies which presented individual participant data, we calculated mean and standard deviation.

## Results

### Search results and study characteristics

1261 articles were identified after entering the search strategy into PubMed and EMBASE. After removing 412 duplicates, 849 records were screened, among which 33 were sought for retrieval, and then 33 were assessed for eligibility. Of these, 24 articles were excluded. The study retrieval process is shown in the PRISMA diagram (Figure 1). Nine studies were included in the systematic review.

The main characteristics of the patients included are summarized in Table 1. 1553 patients were included from the nine studies in the aggregate data sets of all studies. Mean F:M sex ratio was 2.37 (Standard Deviation = 1.03). Studies were from the years 2000–2017. The majority of studies were from the North American continent. Relapsing Remitting MS (RRMS) was the most common MS type.

**Table 1:** Characteristics of included studies.


AUTHOR/YEAR	SETTING	TOTAL NUMBER OF CASES	AGE GROUP IN YEARS (MEAN± S.D)	GENDER RATIO (F:M)	MS PATTERN	DURATION OF MS IN YEARS, MEAN (SD)	MEDIAN EDSS	TREMOR PREVALENCE	SEVERE TREMOR PREVALENCE	TYPE OF TREMOR (% OF TOTAL NUMBER OF CASES)

**Alusi/2001** [8]	Cross-sectional	100	47.2 ± 10.3	1.86	SPMS (63%) PPMS (22%) RRMS (15%)	18.8	6.0 (range 0–9)	58%	15%	Postural (44%) Intention (6%)

**Pittock/2004** [11]	Community- based cohort	200	35 ± 12.4	2.3	RRMS (65%) SPMS (30%) PPMS (5%)	Tremor group 21.9± 12.4; No tremor group 20.4 ± 12.9	3.0 (range 0–9.5)	25%	3%	NA

**Gines/2013** [10]	Cross-sectional	50	45.6 ± 10.8	2.33	RRMS (66%) SPMS (16%) PPMS (6%) CIS (12%)	NA	NA	30%	NA	Postural (20%) Intentional (20%), Simple kinetic (6%) Mixed postural and action (12%)

**Soto/2013** [14]	Cross-sectional	192	NA	NA	NA	NA	NA	53%	NA	Action tremor

**Rinker/2015** [12]	Cross-sectional	552	NA	3.54	RRMS (48.9%)	17.1 ± 8.4	NA	45–46.8%	5.5–5.9%	NA

**Walt/2015** [13]	Case control study	27	50 ± 10.8	1.11	SPMS (74%) RRMS (26%)	17 ± 8.2	5.5 (S.D. = 4. 6)	NA	33%	NA

**Silva/2017** [17]	Cross-sectional	208	NA	NA	NA	NA	NA	12.5%	NA	Intention (10.25%)

**Salari/2018** [9]	Cross-sectional	164	36.35 ± 9.357	4.125	RRMS (59.3%) SPMS (33.6%) PPMS (7.1%)	NA	2.732 (SD = 1.78)	68.9%	14%	Action (41.6%) Resting (30.5%) Intention (43.9%)

**Abboud/2019** [7]	Prospective observational study	60	38.3 ± 12.7	1.3	RRMS (63.3%)	11.4 ± 18.2	NA	41.6%	NA	Action hand (41.67%) Resting (5%) Rubral (3.3%)


### Quality of the included studies

A positive response to each of the aforementioned criteria for the respective JBI tools received 1 point. The case-control study scored 6/10 the cohort study scored 7/11 and out of 7 cross-sectional studies, 5 scored 6/8, 2 scored 7/8. The studies’ overall quality was determined to be satisfactory.

### Prevalence, Severity of tremor

Prevalence of tremor in the MS population among studies ranged widely, between 12.5% to 68.9% (Table 1). The prevalence of severe tremor ranged from 3% to 33% (Table 1).

### Type of tremor

Action tremor was the most frequent type reported in 18–64% [7,8,9]. Intention tremor was present in 12–44% [9,10]. Postural tremor was present in 20% [10]. Resting tremor was present in 30% (50 patients) and 5% (3 patients) by Salari et al. and Abboud et al [8,9]. Rubral tremor was reported in two patients [7].

### Site involved

Upper extremities were the most common site, reported in 23.5–36% in the majority of studies [8,10,11,12]. Tremor in the dominant arm was twice as likely to be reported (67.3%; 140 patients) as in the non-dominant arm (32.7%; 68 patients) [12]. Head and neck tremor was the most common site in one study [13]. Leg tremor was reported only in one study, present in 6% (12 patients) [11]. Tremor of the jaw, tongue or face were not reported [8].

### Predictors of tremor

Age was a significant predictor of tremor in two studies, where younger age of onset of MS was associated with tremor [8,12]. Only one study reported gender as a significant predictor of tremor [12]. The subtype of MS was a significant predictor of tremor in two of the studies [9,11,12]. Patients with secondary progressive MS (SPMS) were more likely to have tremor than the RRMS group [11]. The presence of tremor was strongly associated with EDSS, Incapacity Status Scale (ISS), and Environmental Status Scale (ESS) total scores [11]. However, one study reported no correlation between the EDSS status of these patients and the presence of tremor [14]. None of the studies found a significant relationship between duration of MS and the prevalence of tremor.

### Tremor-related disability

14–37% of patients suffered from incapacitating tremor. There was a strong correlation between tremor and dysarthria, dysmetria and dysdiadochokinesia [9]. Proximal postural tremor caused the greatest tremor-related disability. The tremor-related disability correlated with the maximum tremor severity score. The severity of upper limb tremor highly correlated with ataxic features [8]. The presence of tremor resulting in disability highly correlated with the presence of cerebellar dysfunction [9]. EDSS was independently predicted by the Bain tremor severity levels and substantially linked with cerebellar ataxia scores for the afflicted limb [13]. The majority of tremors were due to infratentorial lesions while the minority were due to spinal lesions [7].

## Discussion

Prevalence of tremor in the MS population ranged between 12.5% to 68.9%. This was variable across hospital-based studies which on average were higher compared to population-based studies [12]. These inconsistent figures were the result of referral bias, with more severely ill MS patients typically recruited by specialized MS centers, and the methods employed to measure tremor prevalence. Despite these estimated numbers, it is still exceedingly challenging to determine the prevalence of tremor in MS because of variable natural history of disease, variability in tremor presentation in terms of types, severity, frequency, evolution, progression.

The most common subtype of tremor was action tremor. The action tremor of MS is the result of damage to the cerebellum or its connections [8]. Resting tremor was detected in 5–30%, which was never reported in Charcot’s observation [7,8,9]. The simultaneous presence of other movement disorders could be a possible reason for this; characterisation of tremor in this context can be aided with the neurophysiological investigations [9]. Likewise, rubral tremor was also detected [8,15,16].

The upper extremities were the most common site in the majority of our studies, followed by head and neck which was the most common site in one study [13]. This is in line with the findings of previous studies [8]. Tremor in the upper extremities is likely more noticeable due to functional demand. Tremor of the tongue, face, or jaw was not reported in the included studies [7].

MS disease duration, or latency from disease onset to the development of tremor, did not determine the degree of associated disability [8]. The tremor-related disability correlated with the maximum tremor severity score [8]. The most severe forms of arm tremor were proximal, causing tremor at the shoulder and were associated with the highest tremor-related disability scores, followed in decreasing order of disability by distal postural/kinetic tremor, isolated intention tremor and finally distal postural tremor, which produced no disability. Conflicting evidence exists regarding an association between the presence of tremor and patient gender as three studies did not find an association while one did [11]. However rigid conclusions cannot be drawn due to lack of large sample size studies. Younger age of patient and SPMS type of MS was associated with the presence of tremor [8,11].

Not surprisingly, tremor-related disability correlated with the severity of tremor. In addition, the severity of tremor correlated strongly with ataxia, dysarthria, dysmetria and dysdiadochokinesia supporting the concept that cerebellar pathology correlates with disability [9]. Severe tremor was a relatively infrequent complication of MS when examined in a community-based cohort.

One limitation of our review is lack of diversity in the patient population either due to lack of data from Asian and African populations or due to exclusion of articles in languages other than English. Other limitations are the small number of patients due to inadequate studies regarding tremor in MS patients.

## Conclusion

Prevalence of tremor was variable across MS population with severe tremor being an infrequent complication. The severity of tremor correlated with increasing disability. The duration of MS did not predict the occurrence of tremor. Upper limb action tremor was the most common, with rare occurrences of resting and rubral tremor. Further studies of tremor in MS patients are warranted to clarify its characteristics and clinical outcomes.

## Abbreviations

CIS: Clinically Isolated Syndrome

NA: Not Available

PPMS: Primary Progressive Multiple Sclerosis

RRMS: Relapsing Remitting Multiple Sclerosis

SPMS: Secondary Progressive Multiple Sclerosis

SD: Standard Deviation
